# An all time low utilization of intrauterine contraceptive device as a birth spacing method- a qualitative descriptive study in district Rawalpindi, Pakistan

**DOI:** 10.1186/1742-4755-10-10

**Published:** 2013-02-09

**Authors:** Amna Khan, Babar Tasneem Shaikh

**Affiliations:** 1 Pakistan Red Crescent Society, Islamabad, Pakistan; 2 Health Systems & Policy Department, Health Services Academy, Chak Shahzad, Park Road, Islamabad 44000, Pakistan

**Keywords:** Contraceptive prevalence rate, Family planning, IUCD, Myths & Misconceptions

## Abstract

**Background:**

Pakistan was among the leading countries in south Asia which started the family planning program in late 50s, forecasting the need to control the population. Despite this early intervention, fertility rate has declined but slower in Pakistan as compared to most other Asian countries. Pakistan has almost a stagnant contraceptive prevalence rate for more than a decade now, perhaps owing to the inadequate performance of the family planning programs. The provision and use of long term contraceptives such as IUCD has always been low (around 2%) and associated with numerous issues. Married women who want to wait before having another child, or end childbearing altogether, are not using any long term method of contraception.

**Methodology:**

A descriptive qualitative study was conducted from May to July 2012, to explore and understand the perceptions of women regarding the use of IUCDs and to understand the challenges/issues at the service provider’s end. Six FGDs with community women and 12 in-depth interviews were conducted with family planning providers. The data was analyzed using the Qualitative Content Analysis approach.

**Results:**

The study revealed that the family planning clients are reluctant to use IUCDs because of a number of myths and misconceptions associated with the method. They have reservations about the provider’s capability and quality of care at the facility. Private health providers are not motivated and are reluctant to provide the IUCDs because of inadequate counseling skills, lack of competence and improper supporting infrastructure. Government programs either do not have enough supplies or trained staff to promote the IUCD utilization.

**Conclusion:**

Besides a well-designed community awareness campaign, providers’ communication and counseling skills have to be enhanced, as these are major contributing factors in IUCD acceptance. Ongoing training of all family planning service providers in IUCD insertion is very important, along with strengthening of their services.

## Introduction

Fertility decline must be the prime purpose of any family planning program besides preserving mother and child health which is of utmost importance for any country to keep health indicators in line with the targets of the Millennium Development Goals. This would only happen when long term contraceptives are promoted and made available to all eligible women looking for long term spacing, through quality family planning services, dedicated work force and state of the art service outlets [1]. Temporary contraceptive methods including condoms are very popular in many developing countries. A condom has a high failure rate (20% with typical use), therefore, it can result in an unwanted or untimely pregnancy. A number of such pregnancies result into multi parity or an unsafe termination of pregnancies and therefore, high maternal morbidity and mortality rates. This may be associated with low use of modern long term contraceptives which provide protection for 4–5 years. The modern Intrauterine contraceptive device (IUCD) is very effective (99%) and an inexpensive family planning method [2]. It is reversible, requires little effort on the part of the user once inserted, and offers 5–10 years of protection against pregnancy. IUCDs wider use would reduce the overall number of unintended pregnancies more than any other method [3].

Pakistan’s population is crossing 180 million whereby the contraceptive prevalence rate (CPR) has been stagnant (around 30%) in Pakistan for more than a decade now. People mostly rely on short term and temporary methods, of which condoms are the most popular one, but with high failure rate because of its incorrect use. Therefore, the number of children per woman in Pakistan is still above 4 with a high (25%) unmet need for contraceptives [4]. This latter may be a contributing factor towards the high maternal mortality ratio in the country as shown in Table 1.

**Table 1:** Comparison CPR & MMR amongst South-Asian countries

**Countries**	**CPR**	**MMR/100,000**
Pakistan	30%	260
Maldives	38%	60
Nepal	47%	170
Bangladesh	54%	240
India	54%	212
Sri Lanka	68%	35
Iran	74%	21

Source: World Development Indicators 2012. World Bank, Washington DC.

**Figure 1:**
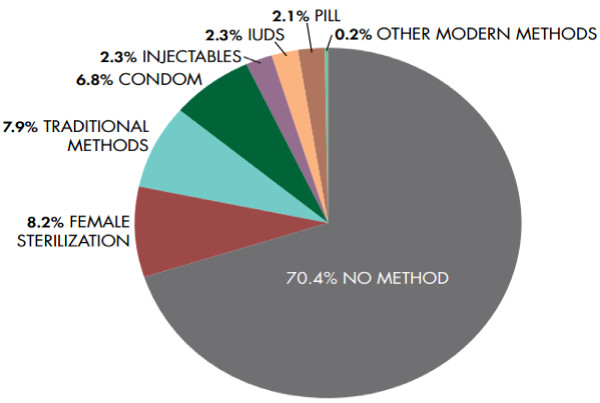
**Current use of different contraceptives in Pakistan. *Source: NIPS & Macro International. PDHS 06-07, Islamabad: 2008.***

A majority of married Pakistani women are not using long term contraception despite the demand. The most common methods used by currently married women are the withdrawal and the rhythm method, condoms or female sterilization, as shown in Figure 1[4].

Family Planning programs have not succeeded in achieving their goals of controlling growth rate of population because they have targeted women who already have had 4–5 children. Temporary hormonal methods of contraception have their own side effects which have created fears among the users. Moreover, the lack of counseling skills among the health providers consulted by the women for child spacing has appeared to further aggravate the situation. To improve contraceptive prevalence rate (CPR) and to control the fertility rate in Pakistan, the reasons for low utilization of long term methods (LTMs) of contraception need to be explored. There might be numerous factors on both sides which may have led to this all time low use of IUCDs: users’ perceptions about the IUCDs and the providers’ bias or other service/supply related issues.

### Study methodology

Qualitative research seeks to understand and interpret the information about the “human” side of an issue i.e. behaviors, beliefs, opinions, emotions and relationships [5]. We used a descriptive qualitative study design to explore and understand the perceptions of women regarding the utilization of IUCDs and to understand the challenges and issues faced by the service providers. This approach helped us to study the different perspectives, conflicting attitudes and experiences in relation to the research topic in depth.

This study was conducted in Tehsil Gujar Khan, one of the largest tehsils of Punjab, in District Rawalpindi, with approximately 73,000 inhabitants. It has one Tehsil Headquarters Hospital, 3 Rural Health centers, several Basic Health Units and many private clinics and maternity homes. The study participants were selected through purposive sampling in 2 Union Councils of the tehsil, one purely rural and the other near the main GT Road, relatively urban. The entire study was completed over a three month time period starting from May to July, 2012.

The study participants were divided into two groups: the permanently residing married women seeking family planning services, particularly those who have a need for long term family planning method; and the service providers providing family planning services in the study area. Pregnant women and women suffering from severe illness were excluded on health grounds.

Data was collected through in depth interviews with the family planning providers and focus group discussions with the community women. The probes for the in depth interviews and focus groups were developed with the help of available literature. Each FGD comprised 8–9 married women, and in all 6 FGDs was conducted. Twelve in depth interviews with family planning service providers were conducted in two Union Councils. Data collection was continued till saturation was reached, that is, when no new information was elicited from the FGD participants. Field notes were taken during or immediately after each interview and discussion session to describe the physical setting and non-verbal communication by the participants. The data was analyzed using Qualitative Content Analysis technique that is defined as “a research method for the subjective interpretation of the content of text data through the systematic classification process of coding and identifying themes or patterns” [6].

Field notes were transcribed in Urdu and then translated into English. The transcribed data were analyzed using steps of content analysis. The transcribed data were read many times to explore and understand different perspectives and experiences of different participants. Analysis started by reducing transcribed text preserving the core meaning of the data. The meaning units identified were then condensed and coded. The codes were grouped into sub-categories and then into categories. The categories were then abstracted into sub-themes and leading to the main theme and that was used to support the conclusions of the study.

### Ethical considerations

A written informed consent was taken from all the study participants. Confidentiality and anonymity was ensured. All the Focus group and interview transcripts were kept in a locked custody of the principal researcher. The study protocol including the written consent form was reviewed and approved by the Institutional Review Board of Health Services Academy, Islamabad, Pakistan.

## Results

The results of our qualitative study fall under three main themes. Factors impeding the IUCD use in the study population have been classified accordingly.

1. Rumors and myths about IUCD as a method of contraception.

2. Inadequate or improper counseling by the service provider to client about IUCD.

3. Inadequate skills of provider related to safe and aseptic IUCD insertion.

### Rumors and myths about IUCD as a method of contraception

Most family planning clients who had never used an IUCD reported a negative impression about the method, mainly because of the fear arising from the rumors and myths they had heard of in their community. In contrast, nearly all IUCD users viewed the method positively. Some of the common misperceptions and misconceptions about IUCD, shared by the prospective family planning clients and thus documented in our study are as follows:

a) “Women who haven’t had children so far, cannot use IUCD”

b) “IUCD act as an abortificient”

c) “The IUCD might travel through the women’s body, maybe to her heart or even to her brain”

d) “IUCD will interfere with sex”

e) “The IUCD may rust in women’s body”

f) “IUCD always causes women to have an ectopic pregnancy”

g) “A woman who has had an ectopic pregnancy history, shouldn’t use IUCD”

h) “IUCD causes infertility”

The health providers on the other hand had misconceptions (which prevail commonly among the community women) regarding the use of IUCD. These are as follows:

a) “It’s hard to get pregnant once women have an IUCD removed”

b) “IUCD spreads infection in all over the body”

c) “IUCD can scar fallopian tubes and after removal woman can’t get pregnant ever”

d) “IUCD is unsafe”

e) “During intercourse, the IUCD can cause pain and discomfort”

f) “IUCD is dislodged during sex”

### Inadequate or improper counseling by the service provider to client about IUCD

While interacting with the community women, it was revealed that they have serious reservations regarding the counseling skills, methodology and interest of the health/family planning provider in discussing matters related to the IUCD.

“Doctors and other service providers advise on the methods which are available with them or for which they feel more convenience in explaining its use”. (FGD 1)

“My neighbor and I went to the same service provider/clinic in the neighborhood and she did not even mention about this method i.e. IUCD”. (FGD 4)

“Our doctor whom we consult for family planning is very competent, but we are not sure why she does not like to talk about IUCDs”. (FGD 5)

Some service providers insisted that they discussed the IUCD in detail with their clients, but many focus-group participants shared that they received information only on pills and injectables. When cross checked with other service providers in the study area, the matter of the fact was somewhat confirmed. All providers seem to have reservations regarding IUCD and therefore they admitted they do not counsel for it, all the time. However, they insisted that counseling is done only on family planning methods which women prefer and that they do not impose any method on them.

### Inadequate skills of provider related to safe and aseptic IUCD insertion

One of the important finding that came out of the focus group discussions is that community women perceive that the family planning staff in the centers or the lady doctors (general practitioners) are not trained in inserting the IUCD. Moreover, it was perceived that their clinic environment was not suitable, and therefore they were reluctant in offering this method.

“Lady doctor does not have the required space, privacy and facility to insert IUCD, therefore she does not offer this method to us”. (FGD 2)

“LHV in government health center has probably never received special training for IUCD insertion and therefore she has never offered any women this method of birth spacing. Traditional birth attendant too are never trained”. (FGD 6)

“The overall cleanliness of a private health center near our house (where we consult lady doctor generally) is so pathetic, that one would never want to have IUCD inserted in that facility. One of my relatives (a woman) had serious infection and therefore I am too much afraid”. (FGD 6)

Many health and family planning service providers, who were trained in IUCD insertion, felt they did not have enough practical experience. Therefore, they avoid inserting the IUCD and do not offer any counseling. If a woman accepts this method, they refer her to a suitably trained service provider, and could potentially lose their client. Some providers seemed hesitant or reluctant to recommend the IUCD, because of its side effects such as irregular menstrual bleeding and pelvic pain.

## Discussion

Our findings identify some of the main reasons for the low utilization of IUCD use in Tehsil Gujar Khan of Rawalpindi district. We have shown that misconceptions, myths and fears about the IUCD discourage its use. Most family planning clients who had never used an IUCD reported a negative impression of the method, mainly because of fear resulting from rumors and myths they had heard. Providers agreed that rumors and myths are the biggest barrier to IUD promotion. However, the health care providers can play an important role in counteracting against those misconceptions. When the providers will do so, clients' attitudes toward the IUCD can improve, and women who opt for the IUCD can become satisfied users. Myths can be stamped out from communities via behavior change communication and social marketing strategy set in context. This approach supports appropriate health seeking behaviors and uptake of IUCD among the community women [7]. Method-specific communication to address myths and misconceptions by giving specific information and testimonials by satisfied clients will help the target audience gain correct information about the methods available to them.

The clients reported that the IUCD was not discussed during their visits, and they often had to request the information. Providers were reluctant to discuss the IUCD because some of them presumed that the clients were not interested and because they did not feel confident in providing it. The lack of confidence was related to a lack of experience in inserting the IUCDs. Method-specific demand creation efforts are effective in increasing accurate knowledge and use of the IUCD, especially when information is conveyed via multiple channels and methodologies and when rumors and myths are addressed directly and candidly [8]. Training is to be comprehensive in nature, and the trainees will be from the public as well as the private sector.

Counseling is a key component of family planning services. The time dedicated to talking with clients can help ensure correct use of and satisfaction with a chosen contraceptive method [9,10]. This study documented that the quality of counseling is perhaps not adequate. During the focus-group discussions, some current or past IUCD users mentioned that very few service providers could successfully dispel the myths associated with IUCD and reassured the clients about the safety of the IUCD. The main reason for discontinuation among the IUCD acceptors, included health concerns (including excessive bleeding, pain and irregular bleeding) which conform to the findings of Bangladesh and Vietnam studies [11]. Nonetheless, the encouraging finding of a recent study is that a large proportion of women (>80%) would still be interested in IUCD, provided they get quality counseling and follow up [12]. Providers’ communication and counseling skills have to be enhanced, as these are major contributing factors in IUCD acceptance. Increasing modern contraceptive method use entails community-wide, comprehensive interventions combining provision of information, life skills, support and access to client-friendly family planning services, especially focusing and countering the negative perceptions of modern contraceptive methods [13].

One quarter of the women in Pakistan have an unmet need for family planning but they are not using any method of family planning, and those who are using some method, either it is not effectively used or it fails and results in an unwanted pregnancy. A woman may then want to abort the pregnancy, and since the services for abortions are not available in the formal health sector for a variety of reasons, she could resort to a clandestine clinic for an abortion, thus putting her life at risk. Such unsafe abortion services may expose the client to a number of complications e.g. septic abortion, infection, perforation of uterus and loss of life. Lack of infrastructure, facilities and resources to cater to the health needs of these clients, is a serious issue which must be addressed by all the stakeholders in women’s health.

The study could not be extended to other areas of the district Rawalpindi due to financial and time constraints, so generalizability remains limited. Moreover, the findings of the study area could be associated with the level of education, demographic and the socio-cultural context which of course is different in other parts of the country.

## Conclusion

Family planning, in general, is the need of the hour to control the population explosion in Pakistan. It is necessary to reach people who need it the most and who need long term, cheap and safe birth spacing methods. There is a need to motivate, educate and make people understand the importance of family planning and usages of IUCD as a long term method, so that it is adopted willfully. Social marketing programs in Pakistan, therefore, must focus on this aspect. Besides training service providers, efforts should be made to provide logistics support and a regular supply of IUCD to ensure an effective and uninterrupted service delivery. Quality of services should be ensured at each level of health care delivery to build client’s trust and to enhance service responsiveness.

## Competing interests

The authors declare that they have no competing interests.

## Authors’ contribution

AK & BTS were involved in conception and design of the study; AK did the analysis and interpretation of the literature as well as the data; and later developed the first draft of the paper; BTS contributed in revising it critically for substantial intellectual content and for adding references. Both authors read and approved the final manuscript.

## Funding support

Authors are thankful to the Health Services Academy for providing the funding for carrying out this important study.
